# The Impact of the Exposome on Alzheimer’s Disease: The Influence of Nutrition

**DOI:** 10.3390/ijms26073015

**Published:** 2025-03-26

**Authors:** Martina Monaco, Carola Torazza, Ernesto Fedele, Massimo Grilli

**Affiliations:** 1 Pharmacology and Toxicology Unit, Department of Pharmacy, School of Medical and Pharmaceutical Sciences, University of Genoa, Viale Cembrano 4, 16148 Genoa, Italy; martina.monaco@edu.unige.it (M.M.); carola.torazza@unige.it (C.T.); ernesto.fedele@unige.it (E.F.); 2 IRCCS Ospedale Policlinico San Martino, 16132 Genoa, Italy

**Keywords:** Alzheimer’s disease, exposome, nutrition, phosphodiesterase (PDE), microbiota, transgenic mouse models

## Abstract

Alzheimer’s disease (AD) is a progressive neurodegenerative disease characterized by cognitive decline, memory loss, and behavioural changes. While genetic predispositions and pathological processes have been the traditional focus, this review highlights the fundamental role of environmental factors, particularly nutrition, within the exposome framework in modulating the risk and progression of AD. The exposome, which includes the totality of environmental exposures in an individual’s lifetime, provides a comprehensive approach to understanding the complex aetiology of AD. In this review, we explore the impact of dietary factors and cyclic nucleotide pathways (cAMP/cGMP) on AD, emphasizing the potential of dietary interventions as therapeutic strategies. We investigate key aspects of how nutrition affects the accumulation of β-amyloid, the aggregation of tau proteins, and neuroinflammation. We also examine the impact of specific nutrients on cognitive performance and the risk of AD. Additionally, we discuss the potential of nutraceuticals with anti-phosphodiesterase activity and the role of various animal models of AD (such as 5xFAD, 3xTg-AD, Tg2576, and APP/PS1 mice) in demonstrating the effects of dietary interventions on disease onset and progression.

## 1. Alzheimer’s Disease and the Exposome

### 1.1. Introduction to AD

Alzheimer’s disease (AD) is a progressive neurodegenerative disorder that predominantly affects older adults and is characterized by cognitive decline, memory loss, and behavioural changes [1]. The global burden of AD is astonishing. According to the latest World Alzheimer Report [2], a new case of dementia develops every three seconds. This alarming statistic underscores the urgent need for effective prevention, treatment, and care strategies. The early stage of AD often manifests as mild cognitive impairment (MCI), where memory deficits exceed normal age-related changes [3]. Like many other neurodegenerative disorders, AD is today also considered a multifactorial disease. Among the multiple factors involved in this pathology, the accumulation of amyloid-β (Aβ), a 42-residue peptide whose production and clearance are finely regulated in the brain, plays a role. Research has demonstrated that impaired clearance of Aβ is a significant factor in AD progression [4]. According to the Aβ hypothesis, soluble aggregates of Aβ peptides, particularly dimers and small oligomers, are highly neurotoxic, disrupting neuronal structure and function [5]. Additionally, Aβ peptides contribute to inflammation [6], tau hyperphosphorylation [7], and, ultimately, cell death [8]. A further hallmark of AD is the formation of neurofibrillary tangles, which are primarily composed of tau protein aggregates. These tangles follow a hierarchical pattern of development in specific brain regions and are closely associated with neuronal loss as the disease advances [9]. Although tau aggregation is widely acknowledged as a pivotal neuropathological hallmark of AD [10], the precise mechanisms that underpin its toxicity remain unclear [11,12]. Another potential contributing factor to the development of AD is neuroinflammation, which plays a pivotal role in both the initiation and progression of the disease. Long-term neuroinflammation can cause cell damage by increasing inflammatory cells, producing reactive oxygen species (ROS), and causing significant changes to DNA [13,14]. Therefore, it seems that inflammation may be a central mechanism in the pathogenesis of AD, prompting the question of whether it is a cause or a consequence of neurodegeneration in this disease [15]. The most probable hypothesis is that there exists a bidirectional relationship between inflammation and AD [16]. While recent immunological therapies have been claimed to possess disease modifying properties, challenges remain regarding their efficacy and safety. These treatments target Aβ deposition, but their impact on disease progression is still debated [17,18]. As a matter of fact, both lecanemab and donanemab, the two anti-Aβ antibodies recently approved by FDA for the treatment of AD, showed marginal, though significant, efficacy on cognitive decline and on activities of daily living, with differences between the placebo and drug-treated populations of 0.45 and 0.7 points, respectively, on the CDR-SB (score range 0–18)in the lecanemab (CLARITY) and in the donanemab (TRAILBLAZER-ALZ 2) clinical trials and 2.91–3.25 points on the iADRS (score range 0–144) in the latter case [18]. Such limited differences have raised many concerns in the scientific community on the clinical relevance of these treatments in the real-world setting. Additionally, administration of these antibodies is accompanied with important side effects (up to 40% incidence) called amyloid-related imaging abnormalities (ARIA), which include brain tissue oedema (ARIA-E) and microbleeds/microhaemorrhage (ARIA-H), representing the major cause of therapy discontinuation [18].

Moreover, emerging evidence highlights the complex interplay between AD and other chronic conditions, such as diabetes, depression, cardiovascular disease, and inflammatory bowel disease [19,20,21,22,23,24,25]. Several new theories have been proposed: chronic neuronal stress [26], the L-isoaspartyl methyltransferase (PIMT) hypothesis [27], the lipid invasion model [28], and the infectious theory [29].

From an etiopathogenic point of view, hereditary factors are significantly linked to the development of late-onset AD; therefore, genetics alone cannot account for the great majority of cases. The fact that many of the comorbidities associated with AD are linked to dysregulated metabolic pathways suggests that lifestyle variables have a role in disease aetiology. In this sense, lifestyle changes such as exercise and diet may interact with inherited susceptibility genes to improve cognition in AD patients [30,31,32,33]. The global AD epidemic demands a multifaceted approach that addresses both biological and lifestyle factors. Continued research into disease mechanisms, the development of innovative treatments, and the promotion of healthy lifestyle interventions is essential to improve the lives of individuals affected by this debilitating condition. Hence, this review aims to shed light on the influence of exposure factors, the “exposome”, on AD, specifically focusing on dietary factors and cyclic adenosine monophosphate (cAMP)/cyclic guanosine monophosphate (cGMP) pathways.

### 1.2. Exposome and AD

The concept of the exposome, which describes the totality of environmental exposures individuals encounter throughout their lifetime, offers a compelling framework for understanding the complex aetiology of AD [34,35]. Traditionally, research on AD has predominantly focused on genetic predispositions and pathological processes [36,37]. Nevertheless, an accumulating body of evidence highlights the substantial influence of environmental factors in modulating disease risk and progression [38]. This notion is particularly relevant for brain health, leading to the term “neural exposome” [39]. Understanding the neural exposome involves recognizing the interplay between various external factors [40]—ranging from air pollution and dietary habits to occupational exposures and psychosocial stressors—and their cumulative impact on brain health [41]. Many studies indicate that long-term exposure to environmental pollutants, such as particulate matter, may contribute to the development of neuroinflammation and oxidative stress, both of which are linked to AD pathology [42,43,44,45,46,47,48,49]. The first studies providing this evidence were conducted on feral dogs naturally exposed to polluted urban environments that showed enhanced oxidative damage, premature presence of diffuse amyloid plaques, and DNA damage in the olfactory bulbs, frontal cortex, and hippocampus [42,43]. The damage of these brain regions occurred in a gradient fashion (olfactory mucosa > olfactory bulb > frontal cortex), indicating the nasal pathway as a key portal entry for pollution. Interestingly, the earliest symptoms in AD include olfactory dysfunctions [44]. Subsequent investigations carried out in mice showed that a prolonged exposure to diesel exhaust particles caused an upregulation of pro-inflammatory cytokines and of the oxidative stress marker malondialdehyde, as well as neuronal morphology alterations in the hippocampus and olfactory bulb [45]. These results were confirmed by other preclinical studies demonstrating that both pre- and postnatal exposure to ambient air pollution (particulate matter diameter of 2.5 µm; PM2.5) induced short-term memory deficits in mice [46]. Furthermore, continuous exposure to PM2.5 caused neuronal loss and tau phosphorylation in the olfactory bulb, in the hippocampus, and in the cortex of 3xTg-AD mice [47]. The link between exposure to air pollution and development of neuroinflammation, oxidative stress, and AD was also shown in several human studies [48]. For instance, post-mortem analyses on human tissues showed that 40% of the exposed urbanites had tau hyperphosphorylation with pre-tangle material, and 51% had diffuse Aβ plaques, together with higher expression levels of genes involved in neuroinflammation and oxidative stress [49].

Adding to this evidence, a comprehensive study of the human exposome using a large-scale multianalyte metabolomics platform revealed that exposure to synthetic chemicals (e.g., persistent organic pollutants) is associated with adverse health outcomes, including neurodegenerative diseases [50]. This underscores the importance of addressing environmental factors in understanding and mitigating AD progression [51]. Furthermore, lifestyle factors, like nutrition and physical activity, are integral components of the neural exposome that can influence cognitive decline and vascular health, thereby indirectly affecting the onset of dementia. It is evident that both aspects are associated with pollution. Indeed, it is acknowledged that food contamination can affect the bioaccumulation of heavy metals, and that outdoor physical activity can expose individuals to particulate matter [50,51]. The complexity of the exposome lies not only in the diversity of exposures but also in the individual variability in response to these factors [52]. Genetic susceptibility can modulate how environmental exposures impact neurological health, directing attention to the importance of personalized approaches in AD research. For instance, variations in genes linked to inflammatory responses may exacerbate the effects of environmental toxins, highlighting the need for integrative models that consider both genetic and environmental influences.

### 1.3. Exposome and Nutrition

**Figure 1:**
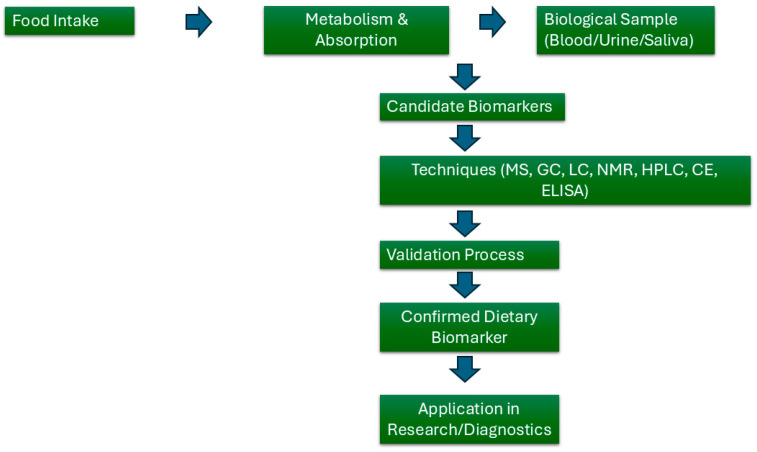
The figure illustrates the common workflow to characterize dietary biomarkers.

Nutrition is of pivotal importance within the exposome framework, as dietary habits exert a significant influence on both individual health outcomes and broader population health trends [53]. Dietary exposures are a principal determinant of health status, given the intricate and varied nature of the foods consumed by humans. Food contains essential macro- and micronutrients, as well as a wide range of non-nutrient compounds, some of which can have either beneficial or detrimental effects on human health [54]. The composition of these compounds is dependent upon several factors, including species, variety, production methods, and food processing. The assessment of the food exposome presents several unique challenges, largely due to the considerable variation in food intake patterns, the diverse chemical composition of food, and the complex interactions between different food components. However, recent technological advances have enabled the identification of dietary biomarkers in biospecimens such as blood, urine, and saliva, thereby providing a more objective approach to the evaluation of dietary exposures (Figure 1) [55].

Mass spectrometry is one of the most widely used techniques for identifying and quantifying dietary biomarkers due to its high sensitivity and specificity. MS can be coupled with other separation techniques such as liquid chromatography (LC-MS) or gas chromatography (GC-MS) to enhance resolution and detection [56,57]. Moreover, MS can be coupled to capillary electrophoresis to separate ions based on their size-to-charge ratio and is particularly useful for analyzing charged metabolites in biological fluids [58].

A universal quantitative analytical method for assessing an organic compound’s purity and spotting potential contaminants is nuclear magnetic resonance (NMR). For nutritional research, NMR can be used in conjunction with chromatography and MS techniques to identify and produce metabolic data [59]. HPLC is a versatile technique that separates compounds based on their interaction with a stationary phase. When combined with detectors such as UV/Vis, fluorescence, or electrochemical detection, HPLC can quantify specific dietary biomarkers [60]. ELISA is an immunoassay technique that uses antibodies to detect specific biomolecules. While less commonly used for dietary biomarkers compared to MS or NMR, ELISA is valuable for detecting large molecules like proteins or peptides [61,62].

The number of identified biomarkers has now surpassed 140 (http://exposome-explorer.iarc.fr, accessed on 25 February 2025), offering an important tool to investigate the influence of specific foods and dietary compounds on the development of chronic diseases such as neurodegenerative disorders [63]. The expanding use of dietary-wide association studies (DWAS) that employ biomarkers holds great promise for enhancing our understanding of the relationship between diet and long-term health outcomes [40]. Overall, nutrition plays a pivotal role in the exposome-related mechanisms from two fundamental perspectives: firstly, as a contributing factor to health, and secondly, as an influential agent in responses to environmental exposures [64,65]. Indeed, dietary patterns and nutrient intake can profoundly impact the body’s resilience against various stresses, including pollutants and toxins [66,67]. For instance, the presence of antioxidants in fruits and vegetables has been shown to mitigate oxidative stress induced by environmental contaminants [68,69,70]. Similarly, a diet rich in omega-3 fatty acids has been observed to reduce inflammatory responses. Consequently, by following a nutritionally balanced and nutrient-dense diet, individuals can enhance their ability to withstand negative health impacts associated with their exposure. Another extremely relevant aspect in the field of exposome-nutrition is the explosion of the supplements market [71]. These products are increasingly enriching the panorama of substances that are taken by the population often without expert monitoring [72]. Finally, the potential of nutrition within the exposome concept must also be analysed based on the characteristics of the subject (age, sex, concomitant diseases, therapies, etc.) [73,74,75].

### 1.4. Nutrition and AD

Recent scientific studies emphasize the connection between dietary habits and cognitive performance. As an example, pre-clinical studies using rodents demonstrated that cognitive decline, typical of aging and neuropsychiatric diseases, is correlated with gut microbiota impairment, which, in turn, can be affected by the quality of dietary habits [76]. Accordingly, Liu and colleagues showed that a high-fiber diet can reduce maternal obesity-induced social dysfunction and cognitive impairment in the offspring of C57BL/6J mice through the modulation of bacterial composition and production of short-chain fatty acids [77]. In line with this finding, several human studies demonstrated that maternal obesity modulates microbiota composition by decreasing the abundance of *Ruminococcus*, *Bifidobacterium*, and *Blautia*, which are associated with impaired cognitive functions in 36-month-old children [78,79,80]. Other studies investigated the impact of a meat-enriched diet on neurodegeneration. In fact, Quan and colleagues [81] demonstrated that a 50 g day^−1^ increment of processed meat is significantly associated with an increased risk of neurodegenerative cognitive disorders, whereas the consumption of fish and poultry seems less harmful.

It is worth noting that, due to the high percentage of people affected by obesity or chronic disease, the use of supplements and diet fads has become an integral part of modern health culture, growing steadily in popularity over the years [72]. These supplements are claimed to have multiple healthy effects on cognitive function, even if they are not supported by robust scientific evidence [72]. They also often lack rigorous clinical testing and may contain unregulated or harmful substances, posing risks of toxicity, adverse reactions, or interactions with prescription medications [72]. Moreover, diet fads that impose extreme caloric restriction or elimination of entire food groups can lead to nutrient deficiencies, metabolic imbalances, or disordered eating patterns. To tackle these challenges, it is essential to prioritize consumer education, healthcare provider involvement, regulatory oversight, and evidence-based research, ensuring individuals make informed health decisions while minimizing the risks linked to diet fads and supplements [72].

The intricate relationship between dietary habits and cognitive function is increasingly recognized as a critical factor in understanding and mitigating AD. A paradigm shift towards emphasizing food quality, rather than simply demonizing specific foods, is essential [82,83]. This necessitates a comprehensive evaluation of food intake, particularly in industrialized nations where overconsumption is prevalent, leading to excessive dietary loads [84,85,86]. At this regard, the notion of Metabolic Food Waste has been established as a metric to assess the implications of Western dietary practices [87]. Overnutrition, resulting from surplus caloric intake, directly contributes to overweight and obesity, highlighting the importance of addressing modifiable risk factors like malnutrition in AD prevention [88,89]. Conversely, targeted nutritional interventions hold promise for neuroprotection. Specific nutrients can modulate inflammation and oxidative stress, key pathological mechanisms in AD [90]. For instance, both ketogenic (KD) and Mediterranean diets exert anti-inflammatory effects by reducing pro-inflammatory cytokine expression, suppressing the COX-2 pathway, decreasing microglia activation, and restoring blood–brain barrier integrity [89,91,92]. Additionally, KD modulates gene expression and affects the gut–brain axis, contributing to its anti-inflammatory effects [92]. Beta-hydroxybutyrate further reduces intracellular levels of reactive oxygen species (ROS) and calcium, while increasing the activities of superoxide dismutase and catalase [93]. Similarly, the Mediterranean diet, rich in antioxidants, lowers postprandial levels of hydrogen peroxide and lipid peroxides [94]. Notably, polyphenols, abundant in this diet, combat neuroinflammation by reducing pro-inflammatory cytokines (IL-1β, IL-6, TNF-α) and inhibiting the NF-κB pathway [95]. Their antioxidant properties neutralize ROS, boost antioxidant enzymes, strengthen the blood–brain barrier, and modulate gut microbiota, all contributing to neuroprotection. A recent study confirms the link between an anti-inflammatory diet and the risk of neurodegenerative diseases [96]. The study analysed the relationship between the Dietary Inflammatory Index (DII), which measures the inflammatory potential of a diet, and the risk of developing dementia. A diet rich in pro-inflammatory foods may increase chronic inflammation in the body, a factor associated with cognitive decline and neurodegenerative diseases [89,97,98]. In contrast, anti-inflammatory foods, such as fruit, vegetables, whole grains, and healthy fats, appear to protect the brain [96]. As a matter of fact, several studies have found a correlation between cognitive function and serum concentrations of key nutrients, such as folate, vitamin B-12, vitamin B-6, and homocysteine [84,85,86]. Among dietary patterns with established neuroprotective effects, the MIND diet, a hybrid of the Mediterranean and DASH diets, has demonstrated slower cognitive decline and reduced AD risk [99]. This diet emphasizes brain-healthy foods while limiting pro-inflammatory items. Higher adherence to the MIND diet is associated with fewer AD-related brain changes, independent of other lifestyle factors [99]. Similar benefits are observed with the Mediterranean and DASH diets, attributed to their ability to reduce oxidative stress and inflammation [100]. The DASH diet, originally designed to lower hypertension, has been associated with brain health due to its encouragement of nutrient-rich foods that help reducing oxidative stress and inflammation [101]. The integration of various diet components into the MIND diet has shown promising results in delaying neurodegenerative diseases and cognitive decline [99,101].

A diet with short- and medium-chain fatty acids (SCFAs, MCFAs, respectively) in animal models of AD (see below) has been shown to lower levels of AD biomarkers (e.g., Aβ peptide), to reduce inflammation, glia activation, oxidative stress, and neuronal loss, and to improve performances in different cognitive tasks [102]. Moreover, it was found that administration of medium-chain triglycerides, which are absorbed and metabolized via medium-chain fatty acids into ketone bodies, to AD patients significantly improved cognitive deficits [103,104].

Additionally, AD shares similarities with other inflammatory conditions linked to the Western lifestyle, where alterations in the gut microbiome and immune pathways have been associated with disease progression [105]. Supporting the link between diet and AD, a study on the App^NL−F/NL−F^ mouse model showed that a high-fat diet (HFD) exacerbates AD pathology by increasing Aβ plaque deposition, promoting oxidative stress, and impairing cognitive function [106]. Furthermore, the 36-month LipiDiDiet trial demonstrated that a multinutrient intervention slowed cognitive decline and brain atrophy and ameliorated memory performance in individuals with prodromal AD, thereby highlighting the role of diet in modifying AD risk [107]. A recent study from Dilmore and colleagues revealed that ketogenic and low-fat diets modulate the human metabolome and microbiome, with the KD particularly showing promise in improving gut microbial profiles and reducing cognitive decline in individuals at risk for AD [108]. This points to the possibility of delaying or treating AD through novel dietary approaches [105]. The health of gut microbiota and the integrity of brain health are interrelated. However, the impact of this relationship on AD is still difficult to establish. A preclinical study using a mouse model of AD demonstrated that Sika deer antler protein bi-directionally modulates the brain and intestinal tract via the tyrosine metabolism pathway, improving the AD symptoms through the activation of the PI3K/AKT/Nrf2 signalling pathway [109]. Probiotics may significantly contribute to mitigating the inflammatory processes that extend from the gut to the brain in the context of AD. By promoting a healthy balance of gut microbiota, these beneficial microorganisms can help reduce inflammation and oxidative stress, potentially lessening the impact of AD on cognitive function and overall brain health [110,111]. In a preclinical study, AD rats that received probiotics (Lactobacillus acidophilus, L. fermentum, Bifidobacterium lactis, and B. longum) had significantly improved spatial memory and reduced oxidative stress biomarkers compared to the AD control group [112]. Moreover, probiotics (a mixture of Lactobacillus acidophilus, Bifidobacterium bifidum, Lactobacillus casei, and Bifidobacterium lactis) improved memory deficits, synaptic and cerebral neuronal injuries, and microglial activation in a mouse model of AD [113]. The mechanism appears to involve inhibition of the caspase-11/caspase-1 pathway and reduction of ALPK1 expression.

### 1.5. cAMP/cGMP Pathways and AD

Cyclic adenosine and guanosine monophosphate (cAMP and cGMP, respectively) are two classical second messengers that are synthesised in cells by different isoforms of adenylyl and guanylyl cyclases (AC; GC) starting from the respective triphosphate substrates [114,115]. While the AC activity is mainly modulated by G-protein-coupled receptors through Gi/Gs protein, the two GC families have different structures, cellular localizations, and mechanisms of activation. In particular, the soluble form of this enzyme represents the heme-based gasoreceptor of the intra/intercellular transmitter nitric oxide (NO), which, in turn, is produced by different NO synthases (namely neuronal, endothelial, and macrophagic NOS), transforming L-arginine into L-citrulline [115]. Moreover, NO can also derive from dietary nitrate, present in several green-leafy vegetables and root vegetables, via the nitrate–nitrite–NO pathway [116]. Interestingly, it has been proposed that flavonoids, a group of natural compounds present in various foods (see below), can enhance NO production through increased expression and activation of endothelial NOS [117]. Similarly, omega-3 polyunsaturated fatty acids (ω3 PUFAs), such as docosahexaenoic acid (DHA) and eicosapentanoic acid (EPA), are able to increase NO, also in this case via endothelial NOS upregulation [118].

It is well established that the two cyclic nucleotides play a crucial role in learning and memory formation/consolidation, basically acting in concert to trigger and maintain the synaptic plasticity process known as long term potentiation (LTP), and, indeed, their modulation has been explored as a potential therapeutic strategy for AD [114,115,119].

Despite being among the most extensively researched therapeutic strategies, modulators of these pathways have exhibited partial outcomes in clinical trials on AD patients, only in part corroborating the preclinical evidence [115,120,121]. It is reasonable that nutrition, as a fundamental element of the exposome, has the capacity to contribute to the homeostasis of cyclic nucleotides. A notable finding in the extant literature is the observation of reduced arginine and citrulline concentrations in the cerebrospinal fluid of individuals diagnosed with AD [122,123,124], which may negatively impact the function of the NO/cGMP pathway. Of note, research has demonstrated that elderly patients afflicted with cerebrovascular disease exhibited enhanced cognitive functions and a reduction in lipid peroxidation levels in response to oral administration of L-Arg [125]. Interestingly, it has been shown that intracerebroventricular administration of arginine to 3xTg-AD mice improved spatial memory acquisition [126]. A recent preclinical study also demonstrated that L-Arg and limonoid supplementation in AD mice reestablished the intracellular GSH levels, reduced oxidative stress, suppressed the secretion of proinflammatory cytokines, and balanced intestinal microflora. These effects were due to the enhancement of communication between gut, pancreas, liver, and brain to produce metabolites that promote host homeostasis [127]. Finally, an oral supplementation with citrulline, which is also a precursor of arginine, was able to improve spatial memory in an AD mouse model, an effect apparently associated with the increase of arginine in the CSF and of NO in the hippocampus [128].

As recently reviewed [129], dietary nitrate can also influence cerebral blood flow and cognitive functions, although there is still no clear evidence, with several clinical trials showing significant beneficial effects while others did not. However, a prospective study on participants of the Rush Memory and Aging Project followed for 4.7 years reported that daily consumption of three green-leafy vegetables (spinach, cabbages, lettuce), containing several bioactive compounds including nitrate, was associated with slower cognitive decline in the higher intake group (median 1.3 servings/day), equivalent to being 11 years younger. Of note, a higher intake of each of the identified nutrients and bioactive compounds (phylloquinone, lutein, folate, α-tocopherol, kaempferol, and nitrate) was associated with a slower cognitive decline [130]. In addition, in a recent prospective study, Rajendra et al. [131] examined the association between the habitual intake of dietary nitrate on older adults of the Australian Imaging, Biomarkers and Lifestyle Study of Aging, cognitively normal at baseline and stratified by *APOE*ε4 status, a polymorphism of the APOE gene known to increase the risk of developing AD. A higher dietary nitrate intake (60 mg/day) was associated with a better language performance in *APOE*ε4 non-carriers, whereas *APOE*ε4 carriers showed better episodic recall memory and recognition memory over 126 months. However, no relationship of dietary nitrate intake with cognitive decline was observed. The same research group also investigated the association between dietary nitrate intake and dementia-related mortality among participants of the Australian Diabetes, Obesity, and Lifestyle study followed over a period of 17 years [132]. They found that participants with the highest intakes of dietary nitrate derived from plants (vegetables, fruits, cereals, herbs, spices, pulses, and nuts; median intake of 98 mg/day) or from just vegetables (median intake of 78 mg/day) had a 57% and 66% lower risk of dementia-related mortality, respectively. As for flavonoids, it remains to be established whether their documented beneficial effects on cognitive functions and in reducing the risk of AD [133] can be attributed, at least in part, to the activation of the NOS/sGC pathway in the cardiovascular and central nervous systems. Regarding ω3 PUFAS, a recent meta-analysis of 21 trials revealed that a one serving/week increment of dietary fish or 0.1 g/day of DHA was associated with a lower risk for dementia and AD, whereas the risk for MCI was lower with an increment of 8 g/day of total PUFAs [134]. However, other studies did not confirm such an association. Therefore, although the existing data favour the idea that an increased consumption of ω3 PUFAS can reduce the risk of cognitive decline and AD, more studies are needed to definitively answer this question and to establish whether this effect is mediated by NO/cGMP.

The importance of cyclic nucleotides in various physiological processes suggests that dietary factors could potentially influence their regulation and signalling [135]. This aspect could be pertinent in determining the actual efficacy of drug treatments in clinical trials and in evaluating their significance in the set endpoints.

### 1.6. Phosphodiesterase Enzymes (PDEs)

The identification of cyclic AMP phosphodiesterase activity in 1962 spurred extensive research into its biochemical characterization, tissue distribution, and physiological functions. Phosphodiesterases (PDEs) are a family of enzymes that catalyse the hydrolysis of phosphodiester bonds in cyclic nucleotides, namely cAMP cGMP, thus terminating their second messenger functions [136]. By regulating the levels of cAMP and cGMP, PDEs play a pivotal role in maintaining cellular homeostasis and influencing metabolic pathways [137]. Subsequent research led to the identification of numerous PDE isozymes, characterized by distinct substrate specificities, regulatory mechanisms, and inhibitor sensitivities. This has culminated in the establishment of a standardized PDE classification system, recognizing eleven distinct PDE families, encoded by 21 genes with multiple variants [138]. Despite sharing a conserved C-terminal catalytic domain, PDEs exhibit variation in their N-terminal regions. Classification of these enzymes is based on C-terminal homology [139]. The substrate specificities exhibited by different PDE families are distinct: some target both cyclic nucleotides (PDE1, 2, 3, 10, 11), while others are selective for cAMP (PDE4, 7, 8) or cGMP (PDE5, 6, 9). They are distinguished by isoform diversity, tissue-specific expression, and subcellular localization, rendering them appealing drug targets. This highlights the potential for targeted interventions also through dietary modifications that can influence specific PDE pathways, ultimately shaping health outcomes. Therapeutic development efforts have yielded numerous selective PDE inhibitors with diverse applications, including the treatment of central nervous system (CNS) diseases such as AD [140].

### 1.7. PDEs and AD

PDE inhibitors have been identified as promising therapeutic agents for the treatment of AD [114,115,119] since, as reported above, cAMP and cGMP signalling are crucial for memory formation and consolidation. These pathways exert significant influence on corticostriatal and hippocampal circuits, impacting both pre- and postsynaptic neurons [141]. Dysregulation of these pathways has been strongly implicated in the pathogenesis of neurodegenerative disorders, including AD [142,143,144,145].

Reduced adenylyl cyclase (AC) activity in the hippocampus and temporal cortex, coupled with increased expression of specific PDE isoforms (e.g., PDE3, PDE4D, PDE7, and PDE8B), has been observed in AD patients [108]. This leads to decreased cAMP-dependent protein kinase A (PKA) activity, as evidenced by reduced cAMP binding to PKA and decreased PKA expression and activity, particularly in severe cases with high amyloid-β (Aβ) burden [146]. Furthermore, cAMP-mediated phosphorylation of Tau at specific sites may contribute to the reduction of neurofibrillary tangles, another pathological hallmark of AD [147,148,149]. Indeed, strategies aimed at elevating cAMP levels, such as PDE4 inhibition, have shown promise in reversing memory deficits in AD [150,151,152,153,154]. These and other observations align with the hypothesis that the cAMP/PKA/cAMP response element-binding protein (CREB) pathway plays a crucial role in mitigating or preventing AD-associated deficits.

As for the cGMP signalling pathway, reduced soluble guanylyl cyclase (sGC) activity has been observed in the superior temporal cortex of AD patient and in reactive astrocytes surrounding Aβ plaques [155,156]. This is accompanied by decreased cGMP levels in the cerebrospinal fluid and platelets of AD patients, which correlates with the severity of memory impairment [143,157]. While some studies have reported increased activity of the NOS/sGC system [158,159], the overall evidence suggests a decline in cGMP signalling in AD. Accordingly, a marked increase in PDE5A mRNA and protein expression has been observed in the temporal and entorhinal cortex of individuals diagnosed with AD [160,161]. In this view, it is worth noting that the PDE5 selective inhibitor vardenafil was able to rescue impaired LTP in functional synapses isolated from parietal cortex samples of AD patients [162]. Recently, a systematic review and meta-analysis of six studies comparing the occurrence of AD between PDE5 inhibitor users and non-users reported a significantly lower risk of developing the disease in the former group [163].

Although inhibitors of cGMP-specific PDEs, namely PDE5 and PDE9, have been shown to exert neuroprotective and memory-enhancing effects in a variety of AD animal models, their translation in clinical trials has not yet yielded consistent results [164,165].

### 1.8. PDEs and Nutrition

A number of naturally occurring compounds present in foodstuffs, including bioactive constituents in a variety of plant extracts, have been shown to exert inhibitory effects on PDE enzymes [166,167]. Furthermore, these compounds have been demonstrated to possess neuroprotective properties that can enhance cognitive function in individuals with dementia and other neurodegenerative disorders [168]. The inhibition of PDE enzymes by natural compounds found in food was initially observed with natural alkaloids such as caffeine [169]. Caffeine, initially known for its bronchodilation properties, was subsequently identified as a non-selective PDE inhibitor, exerting influence over multiple PDE isoforms [169]. This non-selective inhibition contributes to its effects on the CNS and has inspired research into its potential neuroprotective actions, including improvements in cognitive function and support in neurodegenerative disorders. In addition to caffeine, theophylline, another alkaloid present in trace amounts in tea, also functions as a non-selective PDE inhibitor, exhibiting comparable effects on smooth muscle relaxation and bronchodilation [170]. Flavonoids, a group of naturally occurring compounds found in fruits, vegetables, and beverages like tea, have demonstrated PDE inhibitory effects [171]. Key flavonoids, such as quercetin, kaempferol, and catechins act not only as antioxidants but also as modulators of cAMP and cGMP signalling pathways. By selectively inhibiting specific PDE isoforms, they enhance endothelial function, reduce inflammation, and contribute to improved cardiovascular health. Additionally, naringin, a flavonoid present in citrus fruits such as grapefruit, has been investigated as a natural PDE inhibitor. It is particularly noteworthy for its capacity to inhibit cGMP hydrolysis by PDE enzymes, thereby exerting vasorelaxant effects that may ameliorate cardiovascular health and enhance blood flow in the brain, potentially offering protective effects against neurodegenerative conditions [172]. Epigallocatechin-3-gallate (EGCG), a compound present in abundance in green tea, can inhibit all PDE isoforms with some more efficacy on PDE1 and 2. Such a broad inhibitory activity demonstrates significant potential in inducing vasorelaxation, thereby supporting cognitive function and offering neuroprotection against AD [173]. Also, ω3 PUFAs, particularly those derived from fish oil, exhibit inhibitory effects on specific PDE isoforms. This modulation of PDE activity contributes to anti-inflammatory responses and enhances insulin sensitivity, highlighting the critical role of dietary fat quality in metabolic health [174]. Furthermore, the impact of dietary nitrates, abundant in leafy green vegetables and beets, exemplifies the intricate interplay with cGMP signalling, since they are converted to nitric oxide within the body, thereby increasing cGMP levels (see above). While this is a direct effect of a nutrient, it raises questions about the subsequent regulation of PDEs, which might adapt to altered cGMP levels [116]. The implications of PDE activity in relation to nutrition extend to various disease states, including obesity, type 2 diabetes, and cardiovascular diseases, which are also risk factors for AD. Elevated PDE activity has been associated with insulin resistance, indicating that dietary interventions aimed at PDE inhibition may enhance glucose metabolism. For instance, the integration of flavonoid-rich foods into the diet has been shown to bolster cAMP signalling pathways, which may in turn enhance insulin sensitivity and glucose uptake in target tissues [175].

In the domain of cardiovascular health, the interplay between PDEs and nutrition holds particular pertinence. It is widely recognized that chronic inflammation and endothelial dysfunction are common precursors to cardiovascular diseases that, in turn, are regarded as possible risk factors for AD. Intriguingly, olive oil phenols were shown to inhibit PDE3, thus possessing anti-platelet aggregation properties [176]. Consequently, dietary interventions aimed at increasing the intake of PDE-inhibiting foods could serve as non-pharmacological strategies to enhance cardiovascular health. One such example is the Mediterranean diet, which is abundant in fruits, vegetables, whole grains, and healthy fats. This diet has been associated with improved endothelial function and a reduced risk of cardiovascular disease and AD [177,178]. It can be hypothesized that part of these beneficial effects may be attributable to the impact of the Mediterranean diet on PDE activity. The insights gained from understanding the relationship between PDEs and nutrition open doors for novel therapeutic strategies. Nutritional interventions that promote PDE inhibition could play a pivotal role in the prevention and management of chronic diseases. Nevertheless, it is imperative to approach this field with a degree of caution. While dietary supplements that act as PDE inhibitors are emerging, their long-term safety and efficacy require rigorous research.

Furthermore, personalized nutrition represents a promising avenue for maximizing the benefits of PDE inhibition. Genetic variations can influence individual responses to dietary components, suggesting that personalization could enhance the impact of specific diets on PDE activity. Future studies that incorporate genomics and metabolomics into nutritional research could elucidate tailored approaches to harness the power of PDE modulation through diet.

## 2. Transgenic AD Mouse Models and Nutrition

As previously delineated, the dynamic interplay between AD and nutrition could prove pivotal in the development of effective therapeutic interventions for dementia. Consequently, the utilization of animal models of AD is crucial. Indeed, AD mouse models have been widely employed to examine how nutrition affects disease onset and progression [179]. These models exhibit various features of AD, such as amyloid pathology, cholinergic deficits, neurodegeneration, and cognitive impairment [180]. In this section, we will provide an overview of the most frequently used mouse models of AD within the domain of nutrition (Table 1).

### 2.1. Tg2576 Mouse Model

The Tg2576 mouse is a single transgenic model overexpressing the human *APP* Swedish mutation, in which elevated levels of extracellular Aβ can be detected at six months, whereas insoluble plaques start emerging one month later, and behavioural alterations in cognitive domain (e.g., spatial and working memory) manifest at eight to ten months [181,182].

A combination of epigallocatechin-3-gallate, docosahexaenoic acid, and α-lipoic acid effectively reduced Aβ deposition, microglia activation, and memory deficits in the Tg2576 model [183]. Long-term pomegranate supplementation inhibited neuroinflammation and ameliorated the loss of synaptic structure proteins in AD mice [184,185]. Furthermore, dietary supplementation with palm fruits significantly improved learning and memory deficits, enhanced motor coordination, and reduced anxiety, while concurrently decreasing plasma Aβ_40_ and Aβ_42_ levels [186]. Similarly, figs are active on these transgenic mice, reducing oxidative damage and improving cognitive skills [187,188]. Specifically, dietary supplementation with Omani figs for 15 months in Tg2576 mice significantly attenuated oxidative damage, restored antioxidant status, and improved spatial learning and memory, compared to control-fed Tg2576 mice [185,187]. Moreover, it has been reported that adequate intake of docosahexaenoic acid, a n-3 PUFA found predominantly in marine fish that can also be biosynthesized in mammals from linolenic acid, significantly reduced Aβ levels and plaque burden, and modulated APP processing pathways in aged Tg2576 mice [189].

Conversely, a diet deficient in folate, vitamin B6, and B12 accelerated brain amyloidosis in Tg2576 mice [190], and diet-induced hyperhomocysteinemia increased Aβ formation and deposition in the same mouse model [191,192].

### 2.2. APP/PS1 Mouse Model

APP/PS1 mice were generated by combining the human *APP* Swedish mutation with the human *PSEN1* carrying the delta E9 mutation. In this AD mouse model, amyloid plaques start developing at 4–6 months, and deficits across cognitive domains manifest as early as 6 months [181,182,193]. Using this model, it has been shown that a 3-month treatment with pomegranate extract attenuated Aβ plaque load and microgliosis and was able to improve spatial as well as working memory [194]. Pomegranate was also found to inhibit PC12 neuronal death caused by Aβ-induced oxidative stress and to improve learning and memory deficits in mice injected with Aβ_42_ [195]. Similarly, another study in APP/PS1 mice reported that urolithin A, a major metabolite of ellagic acid (derived from the hydrolysis of ellagitannins present in pomegranate and berries), provided neuroprotection by rescuing neurons from apoptosis and by stimulating neurogenesis through the activation of anti-inflammatory signalling pathways, reduced amyloid deposition and microgliosis, and improved spatial learning and memory [196]. Interestingly, metabolomic analysis of serum samples from APP/PS1 mice revealed significant alterations in various metabolic pathways, including energy metabolism, amino acid homeostasis, and membrane lipid metabolism, compared to wild-type mice [197]. In addition, a deficiency in folic acid, which is observed in AD patients [198], has been shown to exacerbate Aβ plaque accumulation in the brain of APP/PS1 mice and to worsen spatial memory but not learning ability [199]. This deficiency also resulted in the diminished expression of specific miRNAs [199] associated with amyloid formation, which may play a crucial role in the progression of neurodegenerative diseases. Moreover, by combining APP/PS1 characteristics with a mutation in cystathionine-beta-synthase, a mouse model with significantly elevated serum homocysteine levels was generated and used to investigate hyperhomocysteinemia that, beside genetic alterations, can result from dietary deficits (e.g., folate, vitamin B_6_ and B_12_) and is considered an AD risk factor [200]. Intriguingly, it was found that Aβ_40_ and Aβ_42_ levels were markedly increased in the brains of female, but not male, mice, thus emphasising a gender effect that deserves further investigations [198]. Finally, in APP/PS1 mice, a restriction in the intake of methionine, an amino acid especially abundant in red meat, has been reported to improve the gut microbiota and faecal metabolites, including SCFAs, able to reduce oxidative stress, enhance energy metabolism, and alleviate Aβ−induced cytotoxicity [201]. Of note, these effects were accompanied with a clear improvement of spatial and non-spatial memory. Remarkably, a faecal microbiota transplantation from transgenic mice with a methionine diet restriction to APP/PS1 mice also reversed the reduction in SCFAs, improving memory and learning scores [201].

### 2.3. 5xFAD Mouse Model

The 5XFAD mouse bears the human *APP* Swedish (KM670/671NL), London (V717I), and Florida (I716V) mutations together with *PSEN1* mutations (M146L/L286V). On average, these mice show Aβ_42_ extracellular deposition by 2 months of age, amyloid plaques at around 6 months, and alterations in different cognitive domains starting at 4–6 months [181,182]. This early-onset transgenic model of AD has been used to study the effects of nutrition on the progression of AD pathology [202].

High-fat diet has been shown to impair energy metabolism in male 5XFAD mice, leading to increased locomotor activity, energy expenditure, and food intake [203]. The microbiome changes caused by a high-fat diet elevated the expression of apoptotic, microglial, and amyloidogenic genes in the hippocampuses of 5XFAD mice. A synbiotic formulation composed of Clostridium sporogenes and xylan enhanced the cognitive performance and spatial memory faculties in this animal model [204]. Interestingly, a Corinthian currant-supplemented diet during the early stages of AD in 5XFAD mice reduced brain oxidative stress through a mechanism involving the Paraoxonase-1 enzyme [205]. In this mouse model, however, the PUFAs ratio did not modify the pathological processes relevant to AD [206]. Oleocanthal, a naturally occurring phenolic secoiridoid isolated from extra-virgin olive oil, has been demonstrated to improve anxiety-like behaviour symptoms and increase sleeping hours in these transgenic mice [207].

### 2.4. 3xTg-AD Mouse Model

This mouse model was developed to replicate tau hyperphosphorylation and neurofibrillary tangle (NTF) formation that, in addition to the amyloid pathology, characterize human AD. Indeed, 3xTg-AD mice contain the *APP* Swedish, the human *PSEN1* (M146V), and the *MAPT* (P301L) mutations and show increased Aβ_42_ levels at 4 months, Aβ plaques around 9 months, NTF at approximately 12 months, and initial cognitive deficits at 4 months [181,182].

In 2024, it was reported that curcumin effectively mitigated cognitive impairments in 3xTg-AD mice subjected to a high-fat, high-sugar diet. This effect was associated with a modulation of the gut microbiota, leading to a reduction in fatty acid synthesis, altered cholesterol metabolism, and suppressed adipogenesis-related pathways in the liver [208]. Resveratrol, another polyphenol, has also demonstrated neuroprotective properties in this mouse model. It has been shown to enhance the effects of exercise training by modulating the toxicity of Aβ oligomers [209]. In this triple transgenic AD mouse model, chronic quercetin administration exhibited neuroprotective effects, and a blueberry polyphenolic extract significantly improved memory performance [210,211]. Conversely, a diet high in saturated fats and deficient in n-3 and n-6 PUFAs has been demonstrated to elevate Aβ concentrations and insoluble tau levels in these AD mice. This diet also decreased cortical levels of drebrin, a postsynaptic marker, suggesting that high-fat consumption combined with low n-3 PUFA intake can promote AD-like neuropathology [212]. These results have been recently confirmed and extended, as it has been shown that the high-fat-diet-induced enhancement of Aβ deposition and tau phosphorylation was associated with microglia damage, neuroinflammation, and marked memory impairment [213]. Consistent with these findings, mice fed a high-fat diet supplemented with branched-chain amino acids exhibited increased tau neuropathology [214]. Moreover, a diet enriched in palmitate and deficient in linoleate intensified oxidative stress and Aβ accumulation within the hippocampus, a process mediated by the activation of NF-κB transcription [215]. In contrast, a diet with high tryptophan levels has been demonstrated to decrease intraneuronal Aβ density in the triple mutant mice. This suggests that augmenting dietary tryptophan intake, and consequently enhancing serotonin neurotransmission, may represent effective strategies for mitigating plaque pathology in AD [216].

**Table 1:** Nutritional and metabolic insights in transgenic AD mouse models.

Transgenic AD Mouse Model	Mechanisms	References
**Tg2576**	Aβ deposition: reduced by epigallocatechin-3-gallate, DHA, α-lipoic acid and dietary supplementation with palm fruit; exacerbated by vitamin deficiencies.	[183,186,191,192]
	Neuroinflammation: inhibited by pomegranate supplementation.	[184,185]
	Oxidative stress: fig extract supplementation reduces oxidative damage.	[187,188]
	Cognitive performance: improved by palm fruit and fig supplementation.	[186,187,188]
**APP/PS1**	Aβ deposition: attenuated by pomegranate extract and exacerbated by folic acid deficiency.	[194,199]
	Neuroinflammation: urolithin A reduces inflammation and stimulates neurogenesis.	[196]
	Oxidative stress: reduced by pomegranate extract.	[195]
	Cognitive performance: improved by urolithin A and microbiota transplantation.	[196,201]
**5xFAD**	Aβ deposition: high-fat diet elevates amyloidogenic gene expression in the hippocampus.	[203]
	Oxidative stress: Corinthian currant-supplemented diet reduces brain oxidative stress via Paraoxonase-1 enzyme.	[205]
	Cognitive performance: improved by a symbiotic formulation composed of Clostridium sporogenes and xylan.	[204]
**3xTg-AD**	Aβ deposition: elevated by high-fat and low PUFAs diets; reduced by high tryptophan levels diets.	[212,215,216]
	Neuroinflammation: resveratrol and quercetin show neuroprotective effects.	[209,210]
	Oxidative stress: a diet enriched in palmitate and deficient in linoleate intensified oxidative stress within the hippocampus.	[215]
	Cognitive performance: improved by blueberry polyphenols.	[211]

## 3. Concluding Remarks

**Figure 2:**
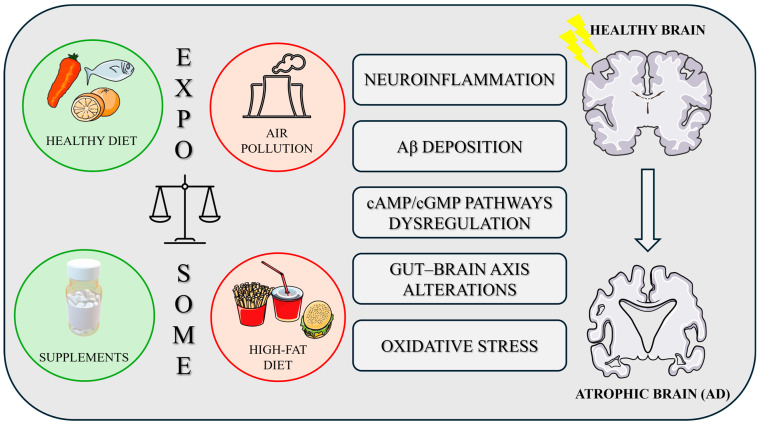
The figure illustrates the comparison between a healthy brain and an atrophic brain associated with AD. It highlights environmental and lifestyle factors influencing brain health, including a healthy diet and supplements as protective measures, versus air pollution and high-fat diets as risk factors for developing AD. Key pathological mechanisms linked to the exposome are shown, including neuroinflammation, gut–brain axis disruption, Aβ deposition, oxidative stress, and altered cAMP/cGMP pathways. The figure was partly generated using Servier Medical Art, provided by Servier, licensed under a Creative Commons Attribution 3.0 unported license (https://smart.servier.com/, accessed on 21 January 2025).

The exposome, particularly the role of nutrition, is crucial in understanding both the causes (aetiopathogenesis) and potential treatments (therapeutic aspects) of AD (Figure 2). This critical understanding is imperative to overcome the current limitations in AD treatment, often referred to as the “therapeutic impasse”.

Research has demonstrated that numerous dietary constituents possess significant modulatory effects that can enhance the efficacy of existing drug-based therapies. Considering the main mechanisms identified, various dietary changes can be tested to decrease Aβ plaque accumulation, regulate the brain–gut axis, lower oxidative stress and neuroinflammation, and alleviate comorbidities such as anxiety and depression. Furthermore, identifying and addressing dietary deficiencies is essential. Finally, recognizing the importance of translating this evidence into clinical practice through well-designed randomized controlled trials, we emphasize the importance of animal models in AD research. Given the promising results observed in AD transgenic mice summarised in this review, which, however, model the very limited percentage of familial AD, naturally occurring models such as SAMP8 mice appear to be increasingly valuable for investigating the impact of nutrition on the pathogenesis of the vast majority of sporadic AD and for developing novel therapeutic/nutraceutical strategies. These animals display a phenotype of accelerated aging and, in our opinion, may be the right model for analysing the impact of nutrition on the development and control of dementia. Indeed, they help to overcome potential bias due to the possibility that genetic variants may alter the microbiome directly, which can result in a specific disease phenotype [217,218].

## Data Availability

Not applicable.
